# Draft genome sequence of *Klebsiella pneumoniae* strain MGC01

**DOI:** 10.1128/mra.00955-26

**Published:** 2026-10-23

**Authors:** Martin Haney, Wei Xu

**Affiliations:** 1 Department of Biomedical Sciences, Marshall University Joan C. Edwards School of Medicine 4034, Huntington, West Virginia, USA; Montana State University, Bozeman, Montana, USA

**Keywords:** *Klebsiella pneumoniae*, sequencing, diabetic wound infection

## Abstract

We report the genome sequence of *Klebsiella pneumoniae* strain MGC01. Whole-genome sequencing generated 9,155,698 high-quality reads, achieving approximately 265-fold coverage. The final assembly comprises 5,159,509 bp with a GC content of 57.55% and 33 contigs. PlasmidFinder identified an IncM1 plasmid replicon, and Kleborate detected the intrinsic β-lactamase gene *bla*SHV-1. This sequence supports molecular epidemiology and pathogenicity studies.

## ANNOUNCEMENT

*Klebsiella pneumoniae* is an encapsulated gram-negative bacillus and one of the most common causes of hospital-acquired infections (1, 2). As an opportunistic pathogen, it is frequently associated with wound and soft tissue infections in immunocompromised individuals.

MGC01 was isolated from a diabetic mouse that developed ulcerative skin lesions with polymicrobial involvement, including *Staphylococcus xylosus*, *Enterococcus faecalis*, and this *Klebsiella* strain (3). The mouse was rendered diabetic using five low-dose streptozotocin injections (3). Infected skin tissue was aseptically excised; the tissue sample mass was approximately 0.5 g. The tissue was homogenized and serially diluted for enumeration of CFU by spot plating on MacConkey agar and cultured overnight at 37°C under static conditions. A single colony was then streaked onto a fresh agar plate and cultured to obtain a pure monoculture (4, 5).

Taxonomic identification was based on 16S rRNA gene amplification and sequencing performed by Genewiz (Azenta Life Sciences, South Plainfield, NJ) prior to whole-genome sequencing. Whole-genome sequencing was not used as the primary method of taxonomic identification; the resulting assembly was consistent with the 16S-based identification. For DNA extraction, an overnight brain heart infusion culture at 37°C was centrifuged (6–9), and the pellet was preserved in DNA/RNA Shield (Zymo Research). Genomic DNA was extracted using the ZymoBIOMICS DNA MiniPrep kit according to the manufacturer’s protocol and quantified using a Qubit 3.0 Fluorometer.

Whole-genome sequencing was performed by Genewiz using Illumina NovaSeq technology. Libraries were prepared with the NEBNext Ultra II DNA Library Prep kit. Genomic DNA was fragmented with a Covaris S220 instrument, followed by cleaning and end-repair. Sequencing libraries were multiplexed and clustered onto a flow cell on the Illumina NovaSeq instrument using a 2 × 150 bp paired-end configuration. Image analysis and base calling were performed via NovaSeq Control Software. A total of 9,254,512 raw reads were generated.

Raw read quality was assessed using FastQC v0.12.1. Reads were trimmed and filtered using fastp v1.0.1 (10–12), resulting in 9,155,698 high-quality reads (2 × 150 bp paired-end). Filtered reads were assembled *de novo* using Unicycler v0.5.1 (13). Contigs shorter than 200 bp were excluded. Assembly quality was evaluated using QUAST v5.3.0 (14, 15) and CheckM2 v1.1.0 (16). Genome coverage was estimated by mapping reads to the final assembly using BWA-MEM v0.7.19 (17–19) and calculating read depth with SAMtools v1.22.1 (20, 21). Prokaryotic genome annotation pipeline (PGAP) v6.11 was used for annotation (22). Plasmid replicons were identified using PlasmidFinder v3.0.3 (23), and antimicrobial resistance and virulence determinants were analyzed using Kleborate v3.2.4 (24). Default parameters were used unless otherwise noted.

The final assembly consisted of 33 contigs, with an N50 of 720,696 bp, a total length of 5,159,509 bp, a GC content of 57.55%, and approximately 265-fold genome coverage. CheckM2 estimated 100% completeness and 0.2% contamination. PlasmidFinder identified a single contig containing an IncM1 plasmid replicon sequence (99.55% identity and 90.66% coverage). Kleborate identified only the intrinsic β-lactamase gene *bla*SHV-1, with no additional resistance or virulence loci detected.

This genomic resource will support future investigations into the pathogenic mechanisms of *K. pneumoniae* in diabetic wound infections (25) and contribute to future genomic analyses and epidemiological investigations.

## Data Availability

Sequencing data for *K. pneumoniae* strain MGC01 are available under BioProject PRJNA1492122 with the corresponding BioSample record SAMN61476706. The whole-genome shotgun project has been deposited at DDBJ/ENA/GenBank under accession number JCENVB000000000. Raw sequencing reads are available under SRA accession number SRR40101950.

## References

[1] Rehman S, Sa-Pessoa J, Buckley C, Lee RL, Wojtania K, Lee A, Lancaster R, Augustine J, Barabas P, Ross C, Zhang X, McCarron JG, Curtis TM, Bengoechea JA. 2026. *Klebsiella pneumoniae* inhibits vasodilation through capsule and T6SS-dependent pathways. Nat Microbiol 11:2170–2186. doi:10.1038/s41564-026-02425-0 PMC13423877 42481698

[2] Chang D, Sharma L, Dela Cruz CS, Zhang D. 2021. Clinical epidemiology, risk factors, and control strategies of *Klebsiella pneumoniae* Infection. Front Microbiol 12:750662. doi:10.3389/fmicb.2021.750662 PMC8724557 34992583

[3] Liu S, Rosen AL, Olson BS, Lu Y, Hickerson S, Kau AL, Hultgren SJ, Caparon MG, Xu W. 2026. ClpX-dependent regulatory pathway exacerbates *Streptococcus pyogenes* pathogenesis in diabetic skin infection. mBio 17:e00486-26. doi:10.1128/mbio.00486-26 PMC13344041 42262120

[4] Xu Wei, Flores-Mireles AL, Cusumano ZT, Takagi E, Hultgren SJ, Caparon MG. 2017. Host and bacterial proteases influence biofilm formation and virulence in a murine model of enterococcal catheter-associated urinary tract infection. NPJ Biofilms Microbiomes 3:28. doi:10.1038/s41522-017-0036-z PMC5673934 29134108

[5] Xu Wei, Chen J, McLellan LK, Flores-Mireles AL, Hunstad DA, Caparon MG. 2025. Host cysteine proteases promote the severity of catheter-associated urinary tract infection and kidney fibrosis. mBio 16:e02161-25. doi:10.1128/mbio.02161-25 PMC12607876 40980878

[6] Merriman JA, Xu W, Caparon MG. 2023. Central carbon flux controls growth/damage balance for *Streptococcus pyogenes*. PLoS Pathog 19:e1011481. doi:10.1371/journal.ppat.1011481 PMC10337930 37384800

[7] Xu Wei, Bradstreet TR, Zou Z, Hickerson S, Zhou Y, He H, Edelson BT, Caparon MG. 2025. Reprogramming aerobic metabolism mitigates *Streptococcus pyogenes* tissue damage in a mouse necrotizing skin infection model. Nat Commun 16:2559. doi:10.1038/s41467-025-57348-x PMC11910614 40089471

[8] Paudel SK, Gannavaram S, Caparon MG, Xu W. 2026. An acetylation-dependent switch underlies host disease tolerance during streptococcal infection. Sci Rep 16:11947. doi:10.1038/s41598-026-42565-1 PMC13069010 41781659

[9] Xu W., Okumura CYM. 2026. Carbon metabolism and niche adaptation in *Streptococcus pyogenes* pathogenesis. mSphere 11:e00668-25. doi:10.1128/msphere.00668-25 PMC13123708 41914738

[10] Chen S, Zhou Y, Chen Y, Gu J. 2018. Fastp: an ultra-fast all-in-one FASTQ preprocessor. Bioinformatics 34:i884–i890. doi:10.1093/bioinformatics/bty560 PMC6129281 30423086

[11] Chen S. 2023. Ultrafast one-pass FASTQ data preprocessing, quality control, and deduplication using fastp. Imeta 2:e107. doi:10.1002/imt2.107 PMC10989850 38868435

[12] Chen S. 2025. Fastp 1.0: an ultra‐fast all‐round tool for FASTQ data quality control and preprocessing. iMeta 4:e70078. doi:10.1002/imt2.70078 PMC12527978 41112039

[13] Wick RR, Judd LM, Gorrie CL, Holt KE. 2017. Unicycler: resolving bacterial genome assemblies from short and long sequencing reads. PLoS Comput Biol 13:e1005595. doi:10.1371/journal.pcbi.1005595 PMC5481147 28594827

[14] Mikheenko A, Saveliev V, Hirsch P, Gurevich A. 2023. WebQUAST: online evaluation of genome assemblies. Nucleic Acids Res 51:W601–W606. doi:10.1093/nar/gkad406 PMC10320133 37194696

[15] Gurevich A, Saveliev V, Vyahhi N, Tesler G. 2013. QUAST: quality assessment tool for genome assemblies. Bioinformatics 29:1072–1075. doi:10.1093/bioinformatics/btt086 PMC3624806 23422339

[16] Chklovski A, Parks DH, Woodcroft BJ, Tyson GW. 2023. CheckM2: a rapid, scalable and accurate tool for assessing microbial genome quality using machine learning. Nat Methods 20:1203–1212. doi:10.1038/s41592-023-01940-w 37500759

[17] Li H, Durbin R. 2010. Fast and accurate long-read alignment with Burrows-wheeler transform. Bioinformatics 26:589–595. doi:10.1093/bioinformatics/btp698 PMC2828108 20080505

[18] Pham M, Tu Y, Lv X. 2023. Accelerating BWA-MEM read mapping on GPUs. ICS 2023:155–166. doi:10.1145/3577193.3593703 PMC10425913 37584044

[19] Houtgast EJ, Sima VM, Bertels K, Al-Ars Z. 2018. Hardware acceleration of BWA-MEM genomic short read mapping for longer read lengths. Comput Biol Chem 75:54–64. doi:10.1016/j.compbiolchem.2018.03.024 29747076

[20] Li H. 2011. A statistical framework for SNP calling, mutation discovery, association mapping and population genetical parameter estimation from sequencing data. Bioinformatics 27:2987–2993. doi:10.1093/bioinformatics/btr509 PMC3198575 21903627

[21] Li H, Handsaker B, Wysoker A, Fennell T, Ruan J, Homer N, Marth G, Abecasis G, Durbin R. 2009. The Sequence Alignment/Map format and SAMtools. Bioinformatics 25:2078–2079. doi:10.1093/bioinformatics/btp352 PMC2723002 19505943

[22] Tatusova T, DiCuccio M, Badretdin A, Chetvernin V, Nawrocki EP, Zaslavsky L, Lomsadze A, Pruitt KD, Borodovsky M, Ostell J. 2016. NCBI prokaryotic genome annotation pipeline. Nucleic Acids Res 44:6614–6624. doi:10.1093/nar/gkw569 PMC5001611 27342282

[23] Carattoli A, Zankari E, García-Fernández A, Voldby Larsen M, Lund O, Villa L, Møller Aarestrup F, Hasman H. 2014. *In silico* detection and typing of plasmids using PlasmidFinder and plasmid multilocus sequence typing. Antimicrob Agents Chemother 58:3895–3903. doi:10.1128/AAC.02412-14 PMC4068535 24777092

[24] Lam MMC, Wick RR, Watts SC, Cerdeira LT, Wyres KL, Holt KE. 2021. A genomic surveillance framework and genotyping tool for *Klebsiella pneumoniae* and its related species complex. Nat Commun 12:4188. doi:10.1038/s41467-021-24448-3 PMC8263825 34234121

[25] T N VV, Premnath M, Stanley JV, Paul N, Mathew J, Radhakrishnan EK. 2025. Whole genome sequencing based prediction of antimicrobial resistance evolution among the predominant bacterial pathogens of diabetic foot ulcer. World J Microbiol Biotechnol 41:161. doi:10.1007/s11274-025-04362-2 40312599

